# The ganglion cell complex as an useful tool in glaucoma assessment

**Published:** 2018

**Authors:** Dana Dascalescu, Catalina Corbu, Valeria Coviltir, Speranta Schmitzer, Mihaela Constantin, Miruna Burcel, Catalina Ionescu, Veronica Strehaianu, Vasile Potop

**Affiliations:** * Oftaclinic Clinic, Bucharest, Romania; ** Clinical Hospital of Ophthalmologic Emergencies, Bucharest, Romania; *** Division of Ophthalmology, Faculty of Medicine, “Carol Davila” University of Medicine and Pharmacy, Bucharest, Romania

**Keywords:** glaucoma, ganglion cell complex, retinal nerve fiber layer

## Abstract

Glaucoma is known as an optic neuropathy prone to progression that determines characteristic not only structural (loss of the ganglion cells as well as their axons) but also functional defects (visual field loss).

**Objective:** To evaluate the possibility of applying ganglion cell complex analysis (GCC) in patients who associate ocular hypertension with tilted disc and marked peripapillary atrophy.

**Methods:** In order to evaluate its components, GCC can be investigated using the Optical Coherence Tomography (OCT) revealing: ganglion cell layer (cells bodies), inner plexiform layer (dendrites and synapses), and nerve fiber layer (axons). Our study included 196 eyes divided into 3 groups: 52 diagnosed with primary open angle glaucoma (POAG), 63 with ocular hypertension (OH), and 81 healthy (normal) eyes (NE). All eyes were submitted to a complete ophthalmologic checkup that involved advanced optic nerve and GCC evaluation.

**Results:** A positive statistically significant correlation was identified between the GCC thickness and the RNFL in all three categories taken into account: R=0,6, p<0,0001 for glaucoma group, R=0,66, p<0,0001 for OH group and R=0,46, p<0,0001 for normal group.

**Conclusions:** GCC has been proved useful for the assessment of the retinal nerve fiber layer (RNFL) in eyes with OH that associate tilted disc or peripapillary atrophy where the optic disc edges might not be certainly determined by the OCT.

## Introduction

Glaucoma is defined as an optic neuropathy that alters the ganglion cells and all their segments: dendrites, cell body, and axon [**1**]. The most important step in glaucoma management is probably the one of the diagnosis [**2**]. Having an early diagnosis is decisive, but usually patients present to the ophthalmologist when their visual acuity starts to decrease and the visual field defect appears. When the visual field defect appears, we can only aim to keep the affection under control and to prevent further visual field loss. In order to save visual function we need to recognize glaucoma prior to this stage and to initiate an early therapeutic plan [**2**-**3**]. 

Studies have shown that the visual field starts to be affected when about 40% of the axons are no longer functional [**4**]. The visual field loss appears only after the RNFL and retinal ganglion cell disturbance [**2**]. In glaucoma, the RNFL defect represents one of the first signs that can be found in a patient [**5**]. However, in a myopic patient with tilted disc and peripapillary atrophy, we cannot determine accurate disc margins and the RNFL analysis might not be exact. In order to confirm our RNFL analysis, we tried to use the macular GCC scan to see if there is any consistency in the two types of scans. 

In glaucoma, ganglion cells usually die by raised intraocular pressure and decreased retrograde axonal outflow. The dendritic arbor is the first to be distressed given the mitochondrial changes, the cell body dies soon after the dendrites, followed by the axon itself [**6**]. 

The ganglion cells can be investigated using the OCT, the scan revealing three different maps that summarize GCC components: nerve fiber layer (NFL), ganglion cell layer (GCL) + internal plexiform layer (IPL) and the GCC (GCL+IPL+NFL). GCC consists of ganglion cell layer that contains cell bodies, internal plexiform layer that contains cell dendrites and synapses with amacrine and bipolar cells, while nerve fiber layer contains cell axons. Knowing the cell death dynamics and the structure of each layer, we understand why the IPL is impaired at the beginning of the condition, followed by the GCL and lastly the NFL [**3**].

## Materials and methods

Our study included 196 eyes from 98 patients divided into 3 groups: 52 eyes with primary open angle glaucoma (POAG), 64 eyes with ocular hypertension (OH), and 80 normal eyes (NE). 

Inclusion criteria included raised intraocular pressure (IOP) in patients who presented glaucomatous optic neuropathy and decreased RNFL with or without visual field damage (POAG group), high IOP in patients with no glaucomatous optic neuropathy, with normal RNFL and unaffected visual field (OH group), normal IOP in patients without glaucomatous optic neuropathy, with normal RNFL and normal visual field (NE group). 

Exclusion criteria for all 3 groups included any macular pathology (epiretinal membrane, diabetic maculopathy, central serous chorioretinopathy, macular degeneration, atrophy or oedema).

All the patients were submitted to a complete ophthalmological examination that began with visual acuity testing, slit lamp examination of the anterior pole, corneal biomechanics investigation in order to establish IOPg, IOPcc, CH and CRF (Ocular Response Analyzer, Reichert, New York), IOP measurements (Goldmann applanation tonometer), ultrasound pachymetry in order to measure central corneal thickness (CCT) (Alcon® OcuScan® RxP Ophthalmic Ultrasound System), gonioscopic examination (Goldmann lens with three mirrors), visual field analyses strategy 24-4 (Humphrey Field Analyzer II Carl Zeiss Meditec Inc, Dublin, California) and finished with fundus examination. 

## Results

The mean IOP in the first group (POAG) was 19,80 (std dev 4,2934), in the second group (OH) group was 19,9821 (std dev 2,9014) along with 17,1125 (std dev 3,4494) in the third group (NE). The mean CCT in the POAG group was 527,8636 (std dev 41,9183), in the OH group was 541,2174 (std dev 28,6968), and in the NE was 549,6458 (std dev 39,3135) (**Table 1**).

**Table 1:** Means of IOP and CCT listed by group

	Mean IOP	Std dev	Mean CCT	Std dev
Primary open angle glaucoma patients	19,80	4,2934	527,8636	41,9183
Ocular Hypertension	19,9821	2,9014	541,2174	28,6968
Normal individuals	17,1125	3,4494	549,6458	39,3135

The mean peripapillary RNFL total in the first group was 71,0204 (std dev 12,5988), in the second group was 101,8413 (std dev 8,1205), and 100,0000 (std dev 7,3976) in the third group. The mean results for peripapillary RNFL inferior in the POAG group were 79,6735 (std dev 18,0236), in the OH group was 123,6032 (std dev 10,7381 ), and in the NE was 123,6173 (std dev 11,4013). The mean peripapillary RNFL superior in the POAG group was 82,6735 (std dev 19,8530), in the OH group was 117,1429 (std dev 13,1858), and in the NE was 119,24 (std dev 14,6910).

The mean GCL+IPL+NFL total in the POAG group was 76,5676 (std dev 8,6234), in the OH group was 103,8254 (std dev 7,0174), and in the NE was 104,6790 (std dev 7,6939). The mean values for GCL+IPL+NFL inferior in the POAG group were 78,2857 (std dev 12,1693), in the OH group was 105,4444 (std dev 7,2061), and in the NE was 90,0278 (std dev 31,5309). The mean GCL+IPL+NFL superior in the POAG group was 78,9714 (std dev 12,0550), in the OH group was 103,1746 (std dev 7,0174), and in the NE was 90,5972 (std dev 29,3165) ) (**Table 2**).

**Table 2:** Means of GCC parameters

	Normal individuals	Ocular Hypertension	Primary open angle glaucoma patients
GCC+IPL+NFL	104,6790	103,8254	76,5676
RNFL	100,0000	101,8413	71,0204

**Fig. 1:**
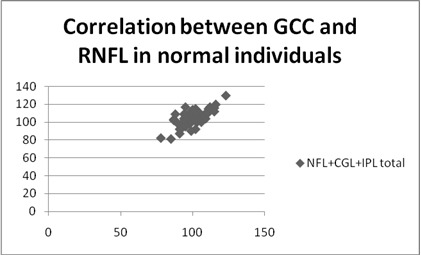
Correlation between GCC and RNFL in normal individuals

**Fig. 2:**
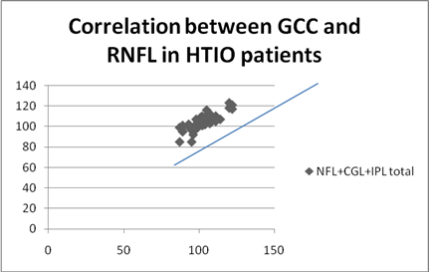
Correlation between GCC and RNFL in HTIO patients

**Fig. 3:**
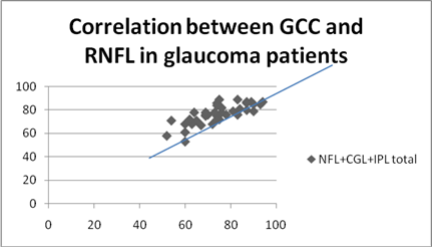
Correlation between GCC and RNFL in glaucoma patients

## Discussions

When facing glaucoma suspects or formerly pronounced glaucomatous patients, we have to take into account many factors such as IOP, rim area, cup/ disc ratio, visual field parameters as well as RNFL and GCC. In some particular circumstances, it is still complicated to issue a glaucoma diagnosis or to illustrate the progression.

As other studies also show, our research established the fact that the measured intraocular pressure was higher in POAG patients and OH group compared to the values objectified within the group consisting of healthy individuals, while CCT was lower in POAG patients than in the OH group and normal individuals [**7**-**9**]. This proves once again that a thin cornea combined with a high IOP represents a risk element for glaucoma.

Our study revealed that the peripapillary RNFL and GCL+IPL+NFL values in glaucomatous eyes are inferior to those in the OH and healthy eyes. An interrelationship between peripapillary RNFL and GCL+IPL+NFL has been found, reinforcing the idea that we might also use GCL+IPL+NFL, along with the RNFL measurement in order to investigate a glaucoma suspect [**10**]. This test may be particularly useful in front of a patient with myopia where specific alterations such as peripapillary atrophy make the papillary margins difficult to identify correctly. 

## Conclusions

When the patient in front of us is a glaucoma suspect, particularly when myopic changes are objectified, we should take into account that one examination could not be sufficient to certify the diagnosis. In eyes with OH that associate tilted disc or peripapillary atrophy where the optic disc limits cannot be undoubtedly set, the GCC could represent a useful tool for the assessment of the NFL. 

Macular GCC thickness can be adopted as a complementary method to discover glaucoma in its preperimetric stage.

**Acknowledgments**

Regarding this paper, all authors have had the same contribution as the first author.
